# Histopathological effects of silver and copper nanoparticles on the epidermis, gills, and liver of Siberian sturgeon

**DOI:** 10.1007/s11356-015-5391-9

**Published:** 2015-09-18

**Authors:** Teresa Ostaszewska, Maciej Chojnacki, Maciej Kamaszewski, Ewa Sawosz-Chwalibóg

**Affiliations:** Division of Ichthyobiology and Fisheries, Faculty of Animal Science, Warsaw University of Life Sciences, Ciszewskiego 8, 02-786 Warsaw, Poland; Department of Biotechnology and Biochemistry of Nutrition, Faculty of Animal Science, Warsaw University of Life Sciences, Ciszewskiego 8, 02-786 Warsaw, Poland

**Keywords:** *Acipenser baerii*, AgNP, CuNP, Nanotoxicology, Epidermis, Gills, Liver, Histopathology

## Abstract

The influence of nanoparticles (NPs) on aquatic environments is still poorly documented. The aim of the study was to determine the effects of silver (AgNPs) and copper (CuNPs) nanoparticles on larval Siberian sturgeon (*Acipenser baerii*) after 21 days of exposure. Acute toxicity of AgNPs on Siberian sturgeon was investigated in a 96-h static renewal study and compared with the toxicity of CuNPs. The AgNPs and CuNPs 96 h mean lethal concentrations (96 h LC50) were 15.03 ± 2.91 and 1.41 ± 0.24 mg L^−1^, respectively. Toxicity tests were done in triplicates for each concentration of AgNPs 0.1, 0.5, 1.5 mg L^−1^ and CuNPs 0.01, 0.05, 0.15 mg L^−1^. The control group was exposed in freshwater. The results indicate that AgNPs and CuNPs exposure negatively influenced survival; body length and mass; and morphology and physiology of the epidermis, gills, and liver of Siberian sturgeon larvae. Fish exposed to AgNPs and CuNPs showed similar pathological changes: irregular structure and pyknotic nuclei of epidermis, aplasia and/or fusion of lamellae, telangiectasis, epithelial necrosis and lifting of the gills, dilation of sinusoidal space, overfilled blood vessels, and pyknotic nuclei of the liver. Fish exposed to CuNPs only demonstrated hyaline degeneration in the gills epithelium and liver. The study shows that CuNPs were more toxic to Siberian sturgeon larvae than AgNPs.

## Introduction

In global industry, the constant pursuit of miniaturization has led to the creation of extremely small particles, the nanoparticles (NPs; 0.1–100 nm). Nanotechnology, the industrial branch that deals with NPs, is still young and promising (Savolainen et al. 2010) and has been developing rapidly for the last 20 years (Farré et al. 2009). Unfortunately, the knowledge about the risks that come with the use of NPs is limited (Christian et al. 2008).

Nanotoxicology analyzes the harmful effects of NPs and their influence on the environment (Handy et al. 2012). The presence of NPs in biosystems may have serious ecological consequences and affect human and animal health (Handy et al. 2008). The most dangerous implications are connected with chronic inhalation and consumption of NPs (Moore 2006). Another serious problem is the accumulation and aggregation of NPs in the aquatic environment, mainly in bottom sediments (Farré et al. 2009). It was described that the sublethal concentration of various nanoparticles for fish ranged from 100 μg L^−1^ to 1 mg L^−1^, while the lethal concentration of nanoparticles reach the milligrams per liter range (Handy et al. 2011). Expected concentrations of NPs in surface waters range from nanograms per liter to low micrograms per liter (Gottschalk et al. 2010; Handy et al. 2011).

However, it was the penetration of NPs into the surface waters that has finally alerted many scientists to focus on aquatic nanotoxicology.

Research on fish (Shaw and Handy 2011; Handy et al. 2011) revealed that NPs are toxic in both high and low concentrations. In fish, signs of chronic toxicity were observed, along with histopathological changes similar to those caused by other xenobiotics (e.g., heavy metals and pesticides) (Boran et al. 2012; Poleksic et al. 2010). The organs most endangered by NPs are the gills, the intestines, and the liver (Handy et al. 2011); but the epidermis may be also affected (Li et al. 2009).

According to Kettler et al. (2014), the main mechanisms of nanoparticle uptake for eukaryotic cells are macropinocytosis, receptor-mediated endocytosis, and phagocytosis. The studies revealed that uptake into non-phagocytic cells depends strongly on NPs size, with an uptake optimum at NPs’ diameter of approximately 50 nm.

Kahru and Dubourguier (2010) classified the NPs of Ag and zinc oxide (ZnO) as “extremely toxic,” C_60_ fullerenes, and CuO as “very toxic”; while other NPs as “toxic” or “harmful.” Silver nanoparticles (AgNPs) are commonly used in various industries (food, textile, paint, or electronics), in different kinds of antibacterial layers (Nowack et al. 2011). AgNPs may also generate the production of oxidants which are responsible for the destruction of the bacteria cell membrane (Kim et al. 2007). AgNPs also affect the protein membranes (OmpA, OmpC, OmpF) causing changes in their structure and functioning, and may affect heat shock proteins (IbpA, IbpB) (Lok et al. 2006; Anas et al. 2012). They also cause changes in 30s ribosomal subunit (Lok et al. 2006). It was observed that AgNPs have proapoptotic and anti-inflammation function as well (Choi et al. 2010).

The annual production of AgNPs is estimated at around 500 t worldwide and grows systematically (Fabrega et al. 2011). Copper nanoparticles (CuNPs) exhibit similar antibacterial properties and are also in broad use, for example, in industrial filter systems (Griffitt et al. 2009). In recent years, NPs were frequently used in aquaculture of fish and seafood for nanofiltration or food packaging (Can et al. 2011; Rather et al. 2011). Even more disturbing is the fact that NPs are used in the production of fish feeds (Handy 2012).

The toxic effect of AgNPs was previously analyzed in zebrafish (*Danio rerio*) (Yeo and Yoon 2009), Japanese medaka (*Oryzias latipes*) (Wu et al. 2010), rainbow trout (*Oncorhynchus mykiss*) (Farkas et al. 2011), crucian carp (*Carassius carassius*), and Eurasian perch (*Perca fluviatilis*) (Bilberg et al. 2011). Toxic effects of CuNPs were observed in zebrafish (Griffitt et al. 2009) and rainbow trout (Al-Bairuty et al. 2013). However, information about the toxicity of these two NPs on other valuable groups of fish such as the sturgeon family (Acipenseridae) is lacking. Sturgeons are on the edge of extinction in their natural habitats, but the ever-increasing demand for their meat and caviar is the cause for the constant production growth in aquacultures located in Asia, Europe, and North America. Despite this, no toxicity tests of AgNPs and CuNPs were conducted on any sturgeon species.

In this experiment, larvae of the Siberian sturgeon (*Acipenser baerii*) were exposed to water solutions of AgNPs (concentrations 0.1, 0.5, 1.5 mg L^−1^), and CuNPs (concentrations 0.01, 0.05, 0.15 mg L^−1^) for 21 days. The aim of the study was to determine how different concentrations of these two NPs affect the larvae survival and development. Histological analysis of the epidermis, the gills, and the liver was conducted.

## Materials and methods

This protocol has been evaluated and approved by the Third Warsaw Local Ethics Committee for Animal Experimentation at Warsaw University of Life Sciences.

### Nanoparticles (AgNPs and CuNPs) used in the experiment

Nanosilver (cat. no 576832, Sigma Aldrich, UK) preparation was based on the manufacturer’s specification for silver nanopowder of particle size <100 nm, surface area 5.0 m^2^ g^−1^, density 10.49 g cm^−1^, and purity of 99.5 %. Nanocopper (cat. no 684007, Sigma Aldrich, UK) preparation was based on the manufacturer’s specification for copper nanopowder of particle size <50 nm, density 8.94 g cm^−1^, and purity of 99.5 %. Silver nanoparticles 50 mg L^−1^ and copper nanoparticles 50 mg L^−1^ were dispersed by sonication in Milli-Q water for 30 min (sonicator, 250 W, 40 kHz, 25 °C; Ultron U-507 Ultron, Poland). After sonication the solutions were filtered through the 200-nm nylon membrane filter (Whatman®, UK).

The distribution and size of nanoparticles were inspected by transmission electron microscopy (TEM) using a JEOL JEM-1220 TE microscope at 80 KeV (JEOL Ltd., Japan), with Morada 11 megapixel camera (Olympus Corporation, Japan). The Zeta potential of the nanoparticles was measured by electrophoretic light-scattering method, using Zetasizer Nano-ZS90 (Malvern, Worcestershire, UK). Each sample was measured after 120 s of stabilization at 25 °C, pH 8.6, in 20 replicates.

### Experimental design

Three-day-old Siberian sturgeon of mean body length 13.30 ± 1.19 mm and body mass 0.016 ± 0.002 g was obtained from the Inland Fisheries Institute in Olsztyn (Poland). The experiments were carried out in the Division of Ichthyobiology and Fisheries, Faculty of Animal Sciences, Warsaw University of Life Sciences. Physical and chemical parameters of water were measured daily (during the acute toxicity test and main experiment). The mean water temperature in experimental tanks was 18.41 ± 0.67 °C, pH 8.6 ± 0.12, and concentration of dissolved oxygen was 8.87 ± 0.35 mg L^−1^. Fish were maintained in 12 h light/12 h dark photoperiods.

Acute toxicity tests lasting 96 h were performed to calculate median lethal concentrations (96 h LC50) of AgNPs and CuNPs. The following concentrations of nanosilver 0, 1, 5, 10, 25, 50 mg L^−1^ and nanocopper 0, 0.5, 1, 2, 4, 6 mg L^−1^ were used. Toxicity tests were done in triplicate for each concentration, 20 fish in each (*n* = 3), in 10 L aquaria. The fish were not fed during the tests. The solutions of silver and copper nanoparticles in experimental aquaria were changed daily. During the 96 h exposure dead fish were counted, and the 96 h LC50 values were calculated using the probit method (Finney 1971). Based on the 96-h LC50 values, sublethal concentrations of nanosilver 0.1, 0.5, 1.5 mg L^−1^ and nano copper 0.01, 0.05, 0.15 mg L^−1^ were used in the experiment.

The fish were exposed in triplicate to each concentration of AgNPs and CuNPs for 21 days under semi-static conditions (80 % of water was changed daily with re-dosing after each change). Control group was exposed in freshwater. The fish were stocked into 21 tanks of 20 L at the density of 2.5 individual per liter.

The fish were fed with *Artemia* sp*.* nauplii (IchthyoTrophic, Poland) ad libitum for the first 5 days, and then commercial sturgeon starter Larva Proactive (Skretting, Norway) was introduced. The following feeding regime was applied: days 1–7, every hour (12 h, 3 % biomass); days 8–14 every 2 h (12 h, 3 % biomass); days 15–21 every 2 h (12 h, 5 % biomass).

### Experimental sampling

On the last day of the experiment, 15 fish (5 fish × 3 tanks) were sampled from each experimental group. The fish were euthanized with MS-222 (ethyl 3-aminobenzoate methanesulfonic acid, 1:5000, pH 7.5 adjusted with NaHCO_3_, Sigma Aldrich, UK). Then they were weighed with 0.001 g accuracy, measured (total body length) with 0.01 mm accuracy, and preserved in Bouin’s solution and 4 % paraformaldehyde for histological and immunohistochemical analyses.

### Histological and immunohistochemical analyses

The fish were subjected to standard histological procedures: they were embedded in Paraplast (Leica Microsystems, Germany); transverse and longitudinal sections were cut into 5-μm-thick slices using microtome Leica RM 2265 (Leica Microsystems, Germany) and stained with hematoxylin-eosin (H&E). Mucin carbohydrates were visualized histochemically (Romeis 1968) with periodic acid–Schiff (PAS), alcian blue 8GX pH 1.0 and pH 2.5, periodic acid–Schiff (AB-PAS). The AB pH 1.0 method was used for staining sulfated glycoconjugates, the AB pH 2.5 method for staining acidic glycoconjugates, and the PAS reaction for visualization of neutral glycoconjugates.

Proliferating cells in the gill and liver were identified using antibodies directed against proliferating cell nuclear antigen according to the method described by Ostaszewska et al. (2008). The gill cell proliferation index was expressed as a number of proliferating cell nuclear antigen (PCNA)-positive cells per number of PCNA-negative cells of gill lamellae. The index was calculated for 20 gill lamellae of 15 fish per experimental group.

Hepatocyte proliferation index was expressed as a number of PCNA-positive cells per number of PCNA-negative cells. PCNA-positive hepatocyte nuclei were counted in liver sections, in 20 fields of 35,000 μm^2^, for 15 fish of each experimental group.

Morphological observations and morphometric measurements (epidermis mucus goblet cell number and area (acidic and neutral), serous goblet cells number, secondary lamellae number, the length of primary and secondary lamellae, hepatocyte surface area, and number of macrophages) were done for 20 randomly selected sections of 15 fish from each experimental group. The mean prevalence of each histopathological parameter was categorized as mild (+, <25 % area of section), moderate (++, 25–50 % area of section) or severe (+++, >50 % area of section).

The measurements were done at ×400 magnification using Nikon ECLIPSE 90i microscope connected with the digital camera Nikon DS5-U1 and the computer image analysis system NIS-Elements AR (Nikon Corporation, Japan).

### Statistical analysis

Fish survival, body mass and length, as well as morphological parameters of the epidermis, gills, and liver were analyses with one-way ANOVA followed by Tukey’s post hoc test (*p* ≤ 0.05) (Statistica 10.0, StatSoft Inc., OK, USA).

## Results

### Characterization of the nanoparticles

**Fig. 1:**
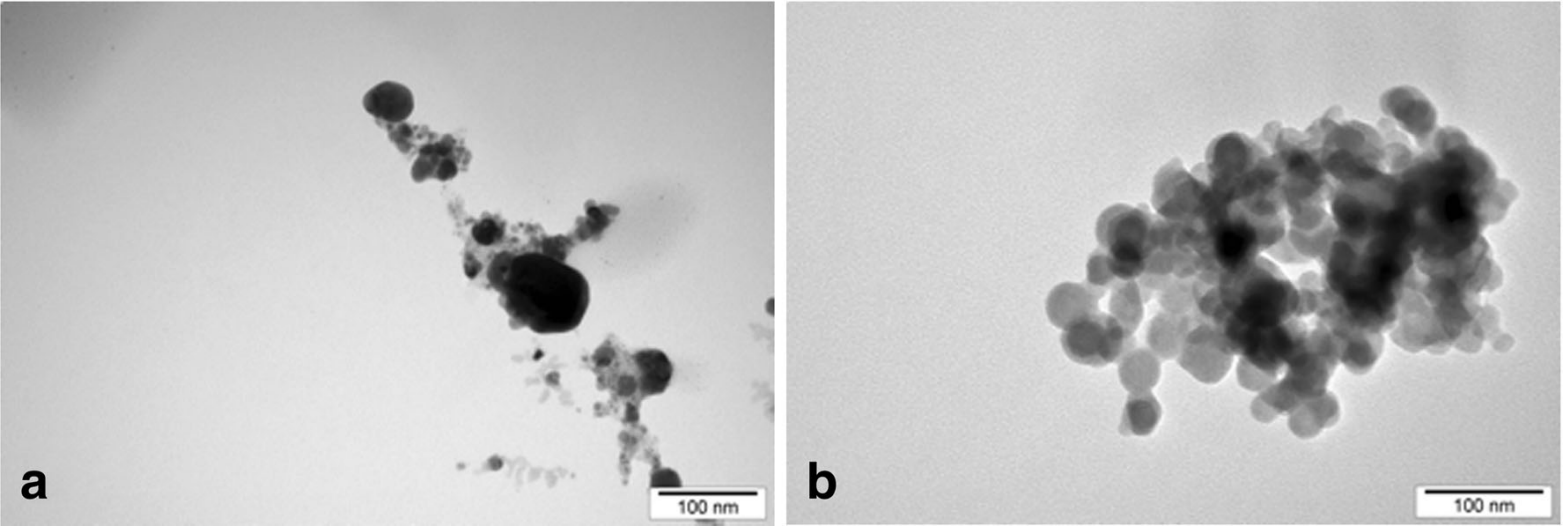
Transmission electron microscopy (TEM) of the nanoparticles: **a** silver nanoparticles and **b** copper nanoparticles

The size of the silver nanoparticles measured with TEM ranged from 4 to 13 nm (average value 8.02 ± 2.49 nm), while the size of copper nanoparticles ranged from 6 to 14 nm (average 10.24 ± 1.99 nm). Silver nanoparticles in the solution formed the aggregates of 235.5 ± 25.1 nm (Fig. 1a), while copper formed the aggregates of 338.0 ± 55.8 nm (Fig. 1b). The zeta potential for nanosilver was −53.6 ± 5.0 mV and for copper, 29.5 ± 0.7 mV.

### Median lethal concentrations (96 h LC50) of AgNPs and CuNPs

**Fig. 2:**
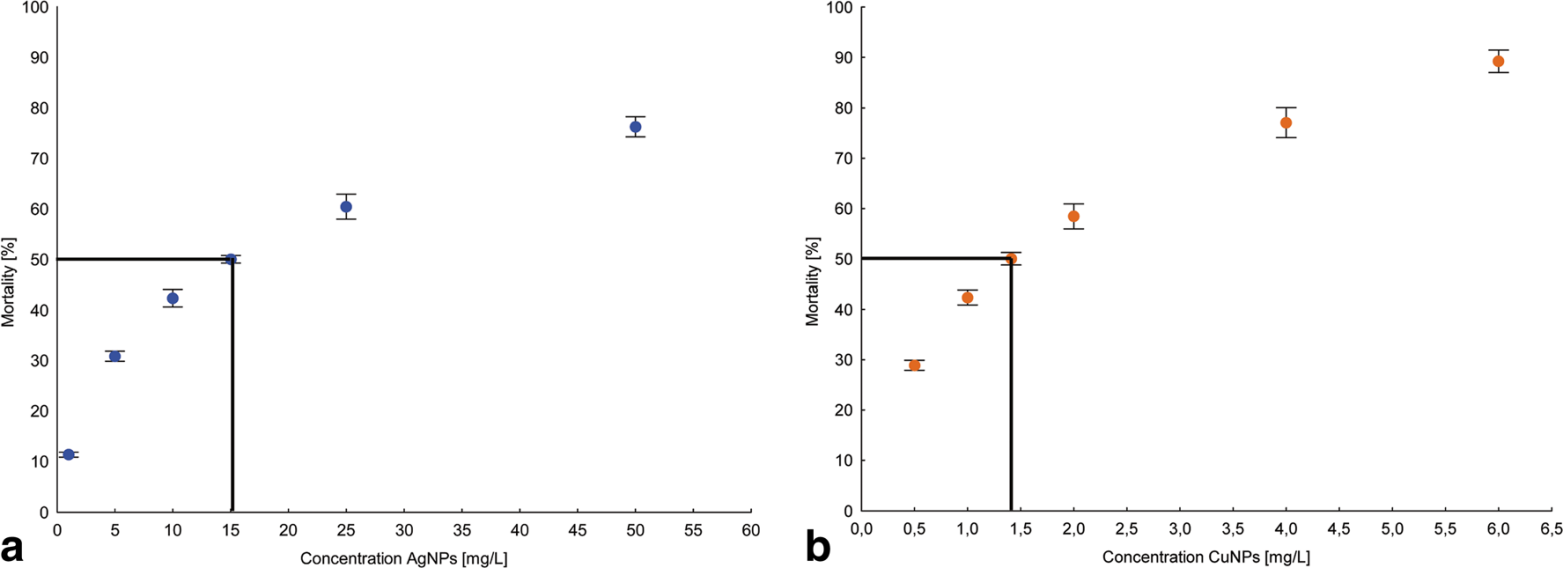
Mortality of Siberian sturgeon during 96 h acute nanosilver (**a**) and nanocopper (**b**) toxicity tests. *Lines* indicate the 96 h LC50 values

The mortality of fish during 96 h acute toxicity tests increased with the increase of AgNPs and CuNPs concentrations (Fig. 2a, b). The concentration of nanosilver causing 50 % fish mortality (96 h LC50) was 15.03 ± 2.91 mg L^−1^, while for nanocopper, 1.41 ± 0.24 mg L^−1^.

### Survival and growth of fish

The control group was characterized by the highest fish survival, body mass, and length. The overall tendency was that all three parameters decreased with increasing NPs concentration (Table 1). Significant reduction of survivability rate and body weight was observed in fish exposed to all concentration of AgNPs. In fish exposed to the highest concentration of CuNPs (0.15 mg L−1), the significant reduction of survivability rate, body weight, and length was found (Table 1).

**Table 1:** Larvae survival (*n* = 3 tanks), body mass and length (*n* = 3), histomorphometry of the epidermis, gills, and liver (*n* = 100), (mean ± SD)

	Parameters	Control	Ag 0.1 mg L^−1^	Ag 0.5 mg L^−1^	Ag 1.5 mg L^−1^	Control	Cu 0.01 mg L^−1^	Cu 0.05 mg L^−1^	Cu 0.15 mg L^−1^
	Survival (%)	77.33 ± 4.16^A^	56.00 ± 5.29^B^	46.67 ± 9.09^BC^	30.67 ± 8.08^C^	77.33 ± 4.16^a^	75.33 ± 5.03^a^	72.00 ± 3.46^ab^	61.33 ± 6.11^b^
	Body weight (g)	0.087 ± 0.015^A^	0.079 ± 0.018^B^	0.067 ± 0.009^BC^	0.049 ± 0.012^C^	0.087 ± 0.015^a^	0.091 ± 0.008^a^	0.066 ± 0.007^b^	0.039 ± 0.003^c^
	Body length (mm)	26.15 ± 2.09^A^	24.65 ± 1.77^AB^	23.95 ± 1.55^AB^	22.40 ± 1.75^B^	26.15 ± 2.09^a^	26.55 ± 1.74^a^	23.53 ± 1.14^ab^	20.80 ± 0.59^b^
Epidermis	Number of MGCs (in 1 mm)	5.4 ± 1.6^B^	8.7 ± 1.7^A^	8.8 ± 1.0^A^	4.8 ± 1.9^B^	5.4 ± 1.6^a^	3.1 ± 1.1^b^	2.9 ± 0.8^b^	2.5 ± 0.8^b^
	Number of SGCs (in 1 mm)	3.3 ± 1.2	5.0 ± 1.8	5.5 ± 2.8	6.1 ± 2.0	3.3 ± 1.2	3.5 ± 2.5	4.1 ± 1.6	5.0 ± 2.7
	Area of MGCs (μm^2^)	220.29 ± 22.96^C^	262.44 ± 18.41^AB^	271.50 ± 20.78^A^	225.14 ± 20.87^BC^	220.29 ± 22.96^a^	152.88 ± 11.49^b^	124.55 ± 11.57^c^	108.40 ± 7.76^c^
	Ratio of acidic MGCs (%)	100 ± 0	100 ± 0	100 ± 0	100 ± 0	100 ± 0^a^	88 ± 4^b^	80 ± 4^c^	25 ± 2^d^
	Ratio of neutral MGCs (%)	0 ± 0	0 ± 0	0 ± 0	0 ± 0	0 ± 0^d^	12 ± 4^c^	20 ± 4^b^	75 ± 2^a^
Gills	Length of primary lamellae (μm)	405.59 ± 35.95^A^	286.54 ± 27.47^B^	273.22 ± 29.43^B^	170.54 ± 12.16^C^	405.59 ± 35.95^a^	281.79 ± 28.27^b^	266.14 ± 33.16^b^	170.19 ± 21.62^c^
	Number of secondary lamellae	16.50 ± 4.20	nd	nd	nd	16.50 ± 4.20^a^	12.33 ± 3.79^b^	nd	nd
	Length of secondary lamellae (μm)	41.65 ± 3.58	nd	nd	nd	41.65 ± 3.58^a^	37.74 ± 2.27^b^	nd	nd
	Proliferative index (%)	20.7 ± 0.7^B^	27.5 ± 1.6^A^	23.0 ± 3.1^AB^	8.8 ± 2.6^C^	20.7 ± 0.7^ab^	25.1 ± 3.3^a^	15.8 ± 3.0^bc^	13.0 ± 3.5^c^
Liver	Area of hepatocytes (μm^2^)	112 ± 17.52^B^	123.54 ± 17.0^AB^	131.44 ± 21.77^A^	79.8 ± 12.56^C^	112 ± 17.52^b^	117.36 ± 14.13^ab^	127.47 ± 22.52^a^	84.9 ± 12.51^c^
	Number of Kupffer cells (in 100 μm^2^)	0.0 ± 0.0^D^	0.059 ± 0.008^C^	0.077 ± 0.008^B^	0.101 ± 0.010^A^	0.0 ± 0.0^d^	0.059 ± 0.009^c^	0.079 ± 0.007^b^	0.103 ± 0.014^a^
	Proliferative index (%)	53.63 ± 4.15^B^	68.43 ± 2.76^A^	66.31 ± 0.70^A^	41.50 ± 2.89^C^	53.63 ± 4.14^bc^	69.01 ± 3.29^a^	61.99 ± 2.50^ab^	46.24 ± 3.75^c^

*Different letters* indicate statistical differences between groups affected by AgNPs (*uppercase letters*) and CuNPs (*small letters*); (*p* ≤ 0.05)

*nd* not detectable

### Histopathology of the epidermis

**Fig. 3:**
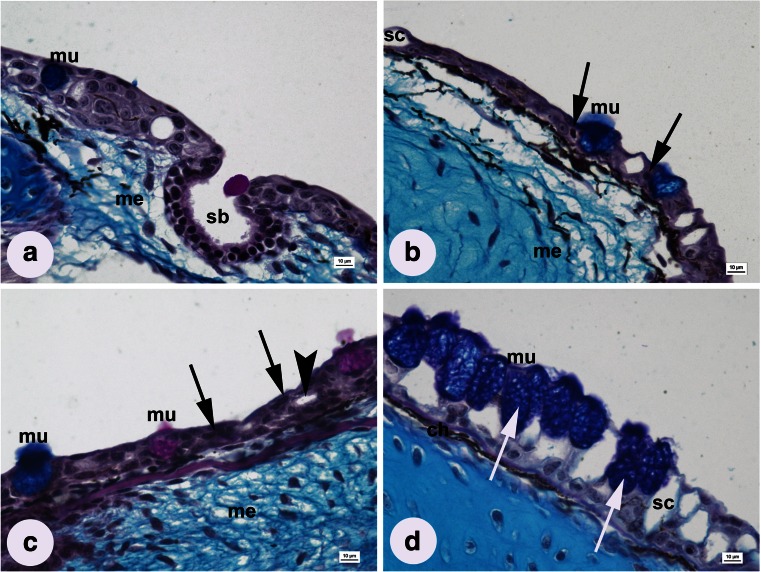
Longitudinal sections of larval epithelium from groups influenced by **a** freshwater (control), **b** 1.5 mg L^−1^ AgNPs, **c** 0.15 mg L^−1^ CuNPs, and **d** 0.5 mg L^−1^ AgNPs. *mu* mucous cell, *sc* serous cell, *sb* sensory bud, *ch* chromatophore, *me* mesenchyme Shrunk cytoplasm (*arrowhead*), pyknotic nucleus (*black arrow*), irregularly shaped mucosomes (*white arrow*); AB/PAS stain; *scale bars* = 10 μm

The epidermis of fish from the control group consisted of regular, stratified squamous, and cuboidal epithelial cells with properly developed nuclei (Table 2). Mucous goblet cells (MGCs) and serous goblet cells (SGCs) were located on the surface of the epidermis. MGCs contained small, regular, round mucosomes and were AB/PAS-positive; SGCs were AB/PAS-negative (Fig. 3a). The average number of MGCs was 5.4 ± 1.6 mm^−1^ of the epidermis (mean cell area 220.29 ± 22.96 μm^2^); the average number of SGCs was 3.3 ± 1.2 mm^−1^ (Table 1).

**Table 2:** Histopathological changes in larvae exposed to AgNPs and CuNPs for 21 days. Lesions were scored based on their severity (− none, + mild, ++ moderate, +++ severe)

Organ	Parameters	Control	Ag 0.1 mg L^−1^	Ag 0.5 mg L^−1^	Ag 1.5 mg L^−1^	Cu 0.01 mg L^−1^	Cu 0.05 mg L^−1^	Cu 0.15 mg L^−1^
Epidermis	Irregular structure	−	+	++	+++	+	+	+++
	Pyknotic nuclei	−	+	+	+++	+	+	++
	Shrunk cytoplasm	−	−	++	+++	−	−	+++
Gills	Aplasia and/or fusion of lamellae	−	+++	+++	+++	+	++	+++
	Epithelial hypertrophy	−	+	++	+++	−	+	++
	Epithelial lifting	−	−	−	+++	−	−	−
	Epithelial necrosis	−	+	+	+++	+	+	+++
	Telangiectasis	−	++	++	+++	−	+	++
	Hyaline degeneration	−	−	−	−	+	+	++
Liver	Kupffer cells	−	+	++	++	+	+	+++
	Dilation of sinusoidal space	−	−	++	+++	−	+	+++
	Pyknotic nuclei	−	−	++	+++	+	+	+++
	Vacuolization of hepatocytes	−	+	+	−	++	+	−
	Shrinkage of hepatocytes	−	−	−	+++	−	−	+++
	Blood cells aggregation	−	−	++	+++	−	+	+
	Hyaline degeneration	−	−	−	−	+	+	+++

Pathological changes in the epidermis were observed in all groups influenced by NPs, especially at the highest AgNPs (1.5 mg L^−1^) and CuNPs (0.15 mg L^−1^) concentrations (Table 2). Irregularly shaped cells were observed, many of them karyopyknotic, and frequently located on the surface of the epidermis. In the middle layers, shrunk cytoplasm led to the occurrence of intercellular spaces. Cell division was common, as were nuclei with irregularly distributed chromatin (Fig. 3b, c).

Compared to the control group, AgNPs concentration of 0.1 and 0.5 mg L^−1^ caused hyperplasia and hypertrophy of epidermal MGCs, while all three CuNPs solutions induced a significant drop of the MGC number and cell area. The 1.5 mg L^−1^ concentration of AgNPs influenced neither of these parameters (Fig. 3 and Table 1). MGCs of fish from the three AgNPs groups were 100 % AB-positive (colored blue), while the number of PAS-positive MGCs (colored magenta) increased with the rise of the CuNPs concentration (Table 1). MGCs of fish affected by AgNPs were characterized by large, irregularly shaped mucosomes (Fig. 3d). The average number of SGCs in all experimental groups was higher than in the control, and these values increased with the growing concentration of both NPs. However, these differences were statistically insignificant (Table 1).

### Histopathology of the gills

**Fig. 4:**
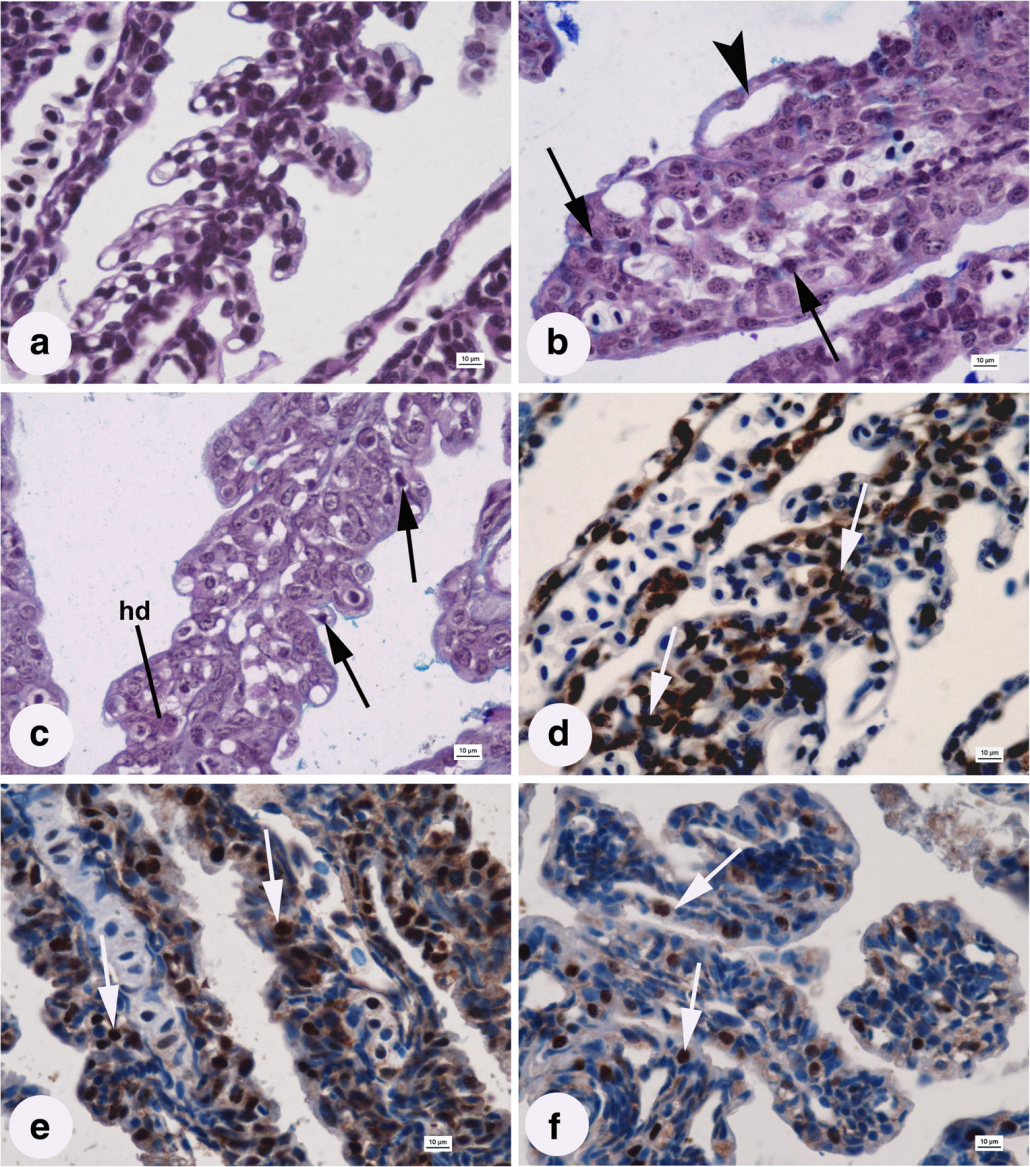
Longitudinal sections of larval gill lamellae from groups influenced by **a** freshwater (control), **b** 1.5 mg L^−1^ AgNPs, **c** 0.01 mg L^−1^ CuNPs (AB/PAS) stain; **d** freshwater (control), **e** 0.5 mg L^−1^ AgNPs, and **f** 0.15 mg L^−1^ CuNPs (immunohistochemical detection of PCNA). *hd* hyaline degeneration. Epithelial lifting (*arrowhead*), pyknotic nucleus (*black arrow*), PCNA-positive nucleus (*white arrows*); *scale bars* = 10 μm

The gills of fish from the control group consisted of well-developed primary lamellae (mean length 405.59 ± 35.95 μm) and secondary lamellae (mean length 41.63 ± 3.58 μm). The average number of secondary lamellae on each primary lamella was 16.50 ± 4.20 (Fig. 4a and Table 1). MGCs were present only on the top of gill arches (pharyngeal side), not on the lamellae.

A series of histopathological changes occurred in the gills of fish exposed to NPs and the most important were non-developed or fused lamellae, which were observed in all experimental groups (Fig. 4b, c). Reduced primary and secondary lamellae length and reduced secondary lamellae numbers were caused by increasing AgNPs and CuNPs concentrations (Table 1). Hypertrophy of the epithelium, observed in all AgNPs groups and the 0.05 and 0.15 mg L^−1^ CuNPs groups, resulted in completely fused secondary lamellae (Fig. 4b). Other anomalies included lifting of the outer epithelial layer, hyaline degeneration (eosinophilic bodies), dilated blood vessels (telangiectasis), and epithelial necrosis (Fig. 4b, c and Table 2).

Compared to the control group, the proliferative index in the gill epithelium was significantly higher in the 0.1 mg L^−1^ AgNPs group (Fig. 4d, e and Table 1) but lower at the highest AgNPs and CuNPs concentrations (Fig. 4f and Table 1).

### Histopathology of the liver

**Fig. 5:**
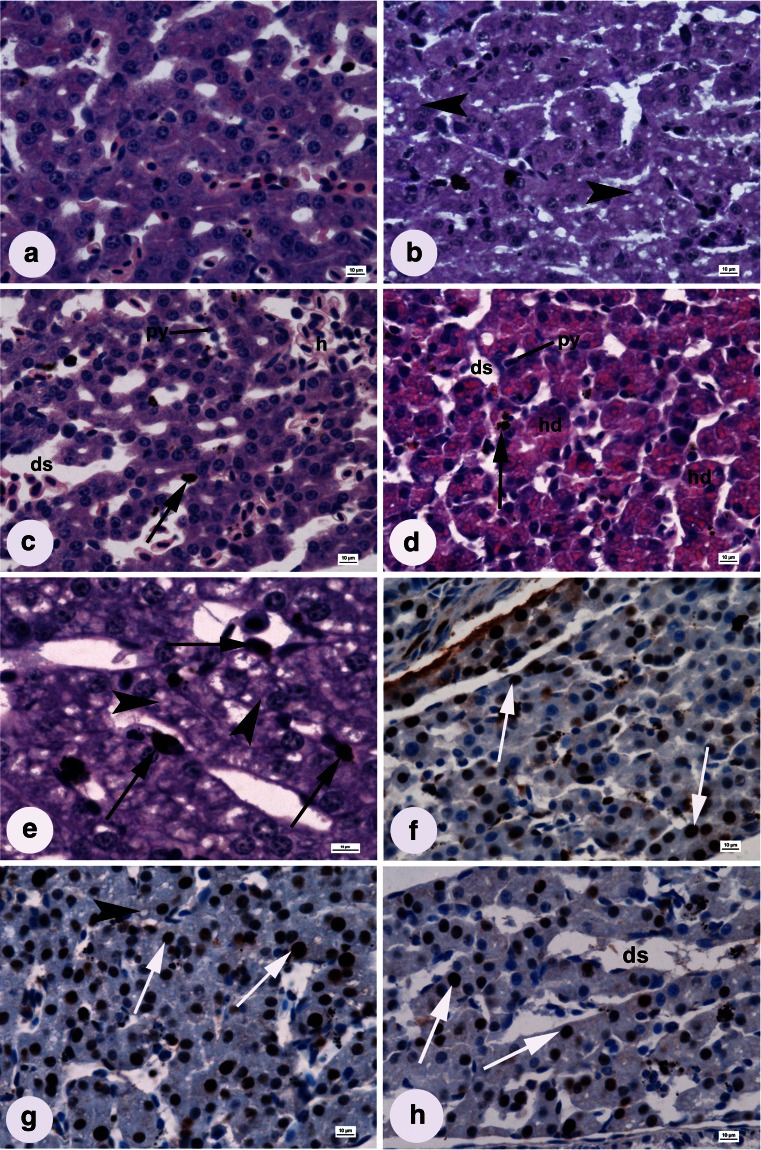
Larval liver sections from groups influenced by **a** freshwater (control), **b** 1.5 mg L^−1^ AgNPs, **c** 0.01 mg L^−1^ CuNPs, **d** 0.15 mg L^−1^ CuNPs, **e** 0.1 mg L^−1^ AgNPs (H&E stain); **f** freshwater (control), **g** 0.5 mg L^−1^ AgNPs, and **h** 0.15 mg L^−1^ CuNPs (immunohistochemical detection of PCNA). *py* pyknotic nucleus, *ds* dilated sinusoid, *h* blood cell aggregation, *hd* hyaline degeneration. Vacuolization (*arrowhead*), Kupffer cell (*black arrow*), PCNA-positive nucleus (*white arrow*); *scale bars* = 10 μm

No signs of histopathological changes were detected in the livers of fish from the control group. Polygonal hepatocytes (mean area of hepatocytes 112 ± 17.52 μm^2^) were regularly located along sinusoids and contained a large, spherical, central nucleus with dispersed chromatin and one or more nucleoli (Fig. 5a and Table 1).

Developmental anomalies in the liver parenchyma of fish affected by NPs included the following: presence of Kupffer cells, karyopyknosis, eosinophilic bodies (hyaline degeneration), dilation of sinusoidal space, blood cell aggregation in blood vessels, hepatocyte vacuolization, and shrinkage of hepatocytes (Fig. 5 and Table 2). Degeneration was more intensive at higher NPs concentrations. Hepatocyte enlargement was caused by vacuolization in the 0.1 and 0.5 mg L^−1^ AgNPs and 0.01 and 0.05 mg L^−1^ CuNPs groups (Fig. 5b). The dilation of sinusoids intensified with increasing NPs concentrations and was additionally enhanced by reduced hepatocyte area (due to shrinkage of cytoplasm) at the highest concentrations of both NPs (Fig. 5c and Table 1). Blood vessels were overfilled with blood cell aggregation in the livers of fish affected by both AgNPs (0.5 and 1.5 mg L^−1^) and CuNPs (0.05 and 0.15 mg L^−1^; Fig. 5 and Table 2), while the eosinophilic bodies were detected only in groups affected by CuNPs (Fig 5d). Pyknotic nuclei were observed in the 0.5 and 1.5 mg L^−1^ AgNPs groups and 0.01, 0.05, and 0.15 mg L^−1^ CuNPs groups (Fig. 4c, d). Kupffer cells were found in all experimental groups (Fig. 5c–e and Table 2).

Comparing to the control group, significantly more proliferating cells were observed in groups affected by 0.1 and 0.5 mg L^−1^ of AgNPs and 0.01 mg L^−1^ of CuNPs (Fig. 5f, g), while significantly lower proliferation occurred in the 1.5 mg L^−1^ AgNPs and 0.15 mg L^−1^ CuNPs groups (Fig. 5h and Table 1).

## Discussion

The results of the present study for the first time report details of the effects of silver and copper nanoparticles on Siberian sturgeon. The values of 96 h LC50 indicate that copper nanoparticles are more toxic to this species compared to nanosilver. According to Kovrižnych et al. (2013) who studied toxicity of 31 nanoparticles to zebrafish, copper and silver were the most toxic. However, these authors evaluated toxicity of nanoparticles of different sizes which makes the comparison of median lethal values obtained in various experiments difficult. According to Hua et al. (2014), the toxicity of nanoparticles depends on their size, with smaller particles being more toxic.

Concentrations of nanoparticles given by Gottschalk et al. (2010) are lower than used in this study. However, it is worth mentioning that the concentration of AgNPs and CuNPs increases every year and soon it is possible that these concentrations will reach sublethal level for aquatic organisms (Griffitt et al. 2007).

Nanoparticles adversely affected fish growth and survival. At the end of the experiment, fish exposed to 0.1, 0.5, and 1.5 mg L^−1^ of AgNPs and 0.05 and 0.15 mg L^−1^ of CuNPs showed lower body mass and length compared to the control group. These results accompanied by low survival indicate the toxic action of AgNPs and CuNPs to Siberian sturgeon larvae. The results of histological analyses revealed also histopathological lesions caused by AgNPs and CuNPs in the epidermis, gills, and liver of sturgeons. The most severe alterations were observed in fish exposed to 1.5 mg L^−1^ of AgNPs and 0.15 mg L^−1^ of CuNPs. Epidermal lesions were found only in the epithelial layer, and their frequency and severity increased with the increase in nanoparticle concentrations. The most commonly observed alterations included irregular structure of epidermal epithelium, contraction of cytoplasm resulting in intercellular spaces, and nuclear pyknosis in external layer of epithelium. Similar lesions in the epidermal epithelium of sterlet (*Acipenser ruthenus* L.) exposed to heavy-metal pollution in the Danube basin were reported by Poleksic et al. (2010). The increase in number of goblet cells and increased mucus secretion are considered the first protective reaction to toxic agents and may temporarily reduce toxic impact (Handy and Maunder 2009). Mucus secretion and swelling of goblet cells in epidermal epithelium were also observed in the present study and were reported by other authors (Smith et al. 2007; Federici et al. 2007) for rainbow trout exposed to other nanoparticles. According to Lee et al. (2012), the increase in the number and size of goblet cells is the reaction to AgNPs.

Serous goblet cells secrete highly proteinaceous content to the epidermal surface. This may provide protection to the fish against various environmental stressors. It has also been suggested that elastin may alter the physical properties of mucous layer by increasing its viscosity, thereby protecting the fish more effectively against chemical damage (Mittal and Agarwal 1977).

Hyperplasia of mucous cells and the increase of serous goblet cell number were the most pronounced lesions in silver-exposed sturgeons. The number of cells secreting acidic mucins (sulfated and carboxylated) increased with the increase of nanosilver concentration; however, at the highest concentration, the number of goblet cells was lower compared to the control. On the contrary, the epithelium of sturgeons exposed to copper showed a reduction of mucous cell number and an increase in abundance of serous cells. The difference in action of AgNPs and CuNPs concerned also the type of mucins secreted by mucous cells. In the epithelium of copper-exposed sturgeons, the number of cells secreting neutral mucins increased with the increase in copper concentration. Fish skin is an important organ participating in osmoregulation and respiration. It also plays a role of the barrier protecting the organism against adverse external conditions. According to Iger and Abraham (1997), who compared the results for various fish species, the number of mucous cells may be an indicator of exposure to stressors. Mucus also contains such compounds as immunoglobulin, lysosome, and lectin that protect fish against infections (Shephard 1994). An increase in the number of mucous cells secreting sulfated and carboxylated mucins is related to the increase in mucus viscosity which improves its protective properties (Kumari et al. 2009). In the present study such an effect was observed in silver-exposed fish. Progressive secretion of neutral mucins instead of acidic ones in fish exposed to copper supports the hypothesis of toxin binding (Perry and Laurent 1993). Reduction of mucous cell abundance at the highest AgNPs concentration and a decrease in the number and area of mucous cells in fish exposed to CuNPs indicate exhaustion of proliferative ability of mucous cells (Poleksic et al. 2010).

In the present study morphometric analysis revealed shortening of primary gill lamellae and fusion or underdevelopment of secondary lamellae. Such an effect was observed at all concentrations of AgNPs, while in fish exposed to CuNPs, shortening of primary lamellae and fusion of secondary lamellae were directly proportional to CuNPs concentration.

Histopathological lesions in fish gills such as epithelial hypertrophy, hyperplasia, lifting, and telangiectasia were described also in other fish species exposed to AgNPs (Wu and Zhou 2013), CuNPs (Al-Bairuty et al. 2013), TiO_2_NPs (Boyle et al. 2013), and other aquatic pollutants (Boran et al. 2012). Shortening and fusion of gill lamellae and epithelial hyperplasia reduce contact of gills with water which results in reduced gas and ion exchange. Bilberg et al. (2010) reported respiratory disturbances and impaired tolerance to hypoxia in Eurasian perch after 24 h of nanosilver exposure. Hypoxic status induced by histopathological lesions was observed in Japanese medaka (Wu and Zhou 2013), and according to these authors it might have resulted in oxidative stress. In the present study nanoparticles of silver and copper caused dilatation of lamellar blood vessels and aggregation of blood cells. According to Martínez et al. (2004), such changes may indicate damage of pillar cells and blood vessels which result in an increase of lamellar blood flow. Siberian sturgeon exposed to AgNPs and CuNPs showed also telangiectasia, epithelial detachment, and epithelial lifting. Epithelial lifting and detachment in secondary lamellae were also observed in Japanese medaka exposed to AgNPs (Wu and Zhou 2013). According to various authors, epithelial lifting usually results from edema of the secondary lamellae (Fanta et al. 2003; Pane et al. 2004). Edema is commonly observed in gills of fish exposed to nanometals. Nanoparticles inhibit ion transport by the branchial Na^+^ and K^+^-ATPase, which results in osmotic imbalance (Shaw et al. 2012; Al-Bairuty et al. 2013). Branchial lesions in Siberian sturgeon caused by AgNPs and CuNPs resulted in cell degeneration and epithelial necrosis, similarly as in Atlantic salmon (*Salmo salar*) exposure also to AgNPs (Farmen et al. 2012).

The hepatic histopathological lesions are often evaluated in toxicological studies and used as markers of environmental pollution (Altinok and Capkin 2007; Dabrowska et al. 2012). The liver shows a high potential of enzymatic degradation of toxic compounds, but it may be itself adversely affected by their high concentrations (Bruslé et al. 1996). Hepatic histopathological alterations in fish exposed to various nanoparticles were already reported by various authors (Govindasamy and Rahuman 2012; Al-Bairuty et al. 2013). Severity of hepatic histopathological alterations in sturgeon increased with the increase in nanoparticle concentrations. The livers of fish exposed to 0.1 and 0.5 mg L^−1^ of AgNPs and to 0.01 and 0.05 mg L^−1^ of CuNPs showed hepatocyte vacuolation and increase in size compared to the control. Similar changes were observed by Hao et al. (2009) in the liver of carp (*Cyprinus carpio*) exposed to TiO_2_NPs and by Al-Bairuty et al. (2013) in the liver of rainbow trout exposed to CuNPs. Abnormal accumulation of triglycerides and other neutral lipids may cause formation of vacuoles in hepatocytes and can be accompanied by pathological lesions such as necrosis (Kelly and Janz 2009). Vacuolation of hepatocytes and the presence of pyknotic nuclei are indicative of the early stages of necrosis (Hao et al. 2009; Al-Bairuty et al. 2013). Govindasamy and Rahuman (2012) found dilation of sinusoid space in the liver of Mozambique tilapia (*Oreochromis mossambicus*) treated with AgNPs. Similar alterations were observed in the present study in sturgeons exposed to 0.5 and 1.5 mg L^−1^ of AgNPs and to 0.05 and 0.15 mg L^−1^ of CuNPs. An increase in sinusoid diameter results from the reduction of hepatocyte size. Hepatocytes of fish exposed to the highest concentrations of both nanoparticles decreased in size and showed karyolysis. Such changes indicate progressive hepatocyte apoptosis and degeneration of hepatic parenchyma caused by the toxic action of nanoparticles (Choi et al. 2010). On the contrary, exposure of rainbow trout to CuNPs caused a decrease in hepatic sinusoid space which indicates redirection of the blood flow to other organs (Al-Bairuty et al. 2013). Hyaline degeneration (storage of the peptides from degraded cells) in the liver, kidney, and gills induced by xenobiotics is a distinct symptom of damage (Altinok and Capkin 2007; Boran et al. 2012). Hyaline degeneration was observed in the liver of carp treated with citrate-capped silver nanoparticles (Lee et al. 2012). In the present study no hyaline degeneration was found in hepatocytes of fish exposed to AgNPs, while distinct symptoms of progressive hyaline degeneration occurred in hepatocytes of fish treated with CuNPs.

The origin and properties of eosinophilic bodies are unknown. These histopathological lesions probably result from the retention of peptide material absorbed from the cytoplasm of damaged cells. Eosinophilic bodies may indicate severe cirrhosis which is suggested by their relation to hepatic necrosis (Costa et al. 2009). The presence of eosinophilic bodies, shrinkage of hepatocytes, nuclear pyknosis, and reduced proliferative potential indicate typical non-specific necrotic lesions.

The hepatic parenchyma of fish treated with nanoparticles showed the presence of sinusoidal Kupffer cells (liver-specialized macrophages), and their frequency was directly related to the concentrations of AgNPs and CuNPs. Macrophages in fish and other animals are responsible for destruction, detoxification, or recycling of endogenous and exogenous materials (Agius and Roberts 2003). Sadauskas et al. (2007) and Priprem et al. (2010) reported the presence of nanoparticles in the cytoplasm of Kupffer cells in the in vitro studies on mice. This finding confirms an important role of macrophages, particularly of Kupffer cells, in scavenging of nanoparticles and explains the increase in their number in the liver of sturgeons exposed to the highest concentrations of AgNPs and CuNPs. However, according to Priprem et al. (2010), hepatocytes and hepatic macrophages may show a different response to the presence of nanoparticles. Macrophages take up the nanoparticles by phagocytosis, while in hepatocyte cytoplasm specific binding takes place, e.g., as SPION (superparamagnetic iron oxide nanoparticles) or with proteins (Priprem et al. 2010). In rat, small granules of AgNPs (autometallographic) were observed in or around hepatocytes (Loeschner et al. 2011). Alterations in hepatocyte cytoplasm observed in the present study suggest that nanoparticles may interact with enzymes and other hepatic proteins affecting antioxidative response and may generate reactive oxygen species (ROS) which may result to oxidative stress leading to atrophy and necrosis.

Participation in replication and repair of DNA are well-known functions of PCNA (Essers et al. 2005). Therefore, the increase in PCNA expression in nuclei of branchial and hepatic cells observed in sturgeons exposed to AgNPs (0.1, 0.5 mg L^−1^) and CuNPs (0.01 mg L^−1^) may be explained as a protective response. On the other hand, lower proliferative index in the gills and liver of fish exposed to the highest concentrations of nanoparticles indicates exhaustion of the proliferative potential which is confirmed by necrotic lesions observed in these organs. A decrease in hepatocyte proliferation rate was also observed in Japanese medaka embryos subjected to hypoxia (Cheung et al. 2012), which suggests the possibility of hypoxic liver injury in sturgeons.

## Conclusions

This study proved, basing on the 96 h LC50 for Siberian sturgeon, that AgNPs and CuNPs indicated toxicity on Siberian sturgeon larvae. Siberian sturgeon exposed to AgNPs shows lower survival, body mass, and length in comparison with the sturgeon exposed to CuNPs. However, the concentration of CuNPs was ten times lower than the concentration of AgNPs. Also, depending on the kind of nanoparticles, the reaction of mucous goblet cells of epidermis varied. Mucous goblet cells of the epidermis in fish exposed to CuNPs displayed lower area and a higher number of cells secreting neutral mucus, which suggest more enhanced body reaction compared to the epidermis mucous goblet cells of fish exposed to AgNPs. However, hyaline degeneration in the gills epithelium and in the liver of fish exposed to CuNPs shows irreversible pathologic alterations. The result of the study shows that during the Siberian sturgeon development, CuNPs are more toxic than AgNPs.
